# Five-Minute Apgar Score and the Risk of Mental Disorders During the First Four Decades of Life: A Nationwide Registry-Based Cohort Study in Denmark

**DOI:** 10.3389/fmed.2021.796544

**Published:** 2022-01-14

**Authors:** Hua He, Yongfu Yu, Hui Wang, Carsten Lyng Obel, Fei Li, Jiong Li

**Affiliations:** ^1^Developmental and Behavioral Pediatric Department and Child Primary Care Department, Xinhua Hospital Affiliated to Shanghai Jiao Tong University School of Medicine, Shanghai, China; ^2^Ministry of Education-Shanghai Key Laboratory of Children's Environmental Health, Xinhua Hospital Affiliated to Shanghai Jiao Tong University School of Medicine, Shanghai, China; ^3^Department of Biostatistics, School of Public Health and the Key Laboratory of Public Health Safety of Ministry of Education, Fudan University, Shanghai, China; ^4^Department of Clinical Epidemiology, Aarhus University, Aarhus, Denmark; ^5^Department of Public Health, Aarhus University, Aarhus, Denmark

**Keywords:** 5-min Apgar score, mental disorders, childhood, early adulthood, cohort study

## Abstract

**Objectives:**

The associations of long-term risks of the full spectrum of mental disorders with clinically reassuring but suboptimal score range 7–9 remain unclear. This study investigated these associations during up to 38 years of follow-up.

**Methods:**

In a nationwide cohort study of 2,213,822 singletons born in Denmark during 1978–2015, we used cox regression to estimate the hazard ratio (HR) of mental disorders with a 95% CI.

**Results:**

A total of 3,00,679 (13.6%) individuals were diagnosed with mental disorders. The associations between suboptimal Apgar score 7–9 and mental disorders differed by attained age. In childhood (≤ 18 years), declining Apgar scores were associated with increased risks of overall mental disorders with HRs (95% CI) of 1.13(1.11-1.15), 1.34 (1.27–1.41), and 1.48 (1.31–1.67) for Apgar scores of 7–9, 4–6, and 1–3, respectively, compared with a score of 10. A dose-response association was seen even within the score range from 9 to 7 (HR 1.11 [95% CI: 1.08–1.13], 1.14 [1.10–1.18], and 1.20 [1.14–1.27], respectively). Of note, individuals with scores of 7–9 had increased risks of organic disorders (HR: 1.27, 95% CI: 1.05–1.53), neurotic disorders (HR: 1.07, 95% CI: 1.03–1.11), and a wide range of neurodevelopmental disorders, such as intellectual disability (1.87, 1.76–1.98), childhood autism (1.13, 1.05–1.22) and attention deficit hyperactivity disorder (1.10, 1.06–1.15). In early adulthood (19–39 years), suboptimal Apgar scores 7–9 were not associated with the risks of overall and specific mental disorders.

**Conclusion:**

Infants born with clinically reassuring but suboptimal 5-min scores 7–9 are at increased risks of a wide spectrum of mental disorders in childhood.

## Introduction

The Apgar score is based on five components (skin color, heart rate, reflex irritability, muscle tone, and respiration). Each item is scored from 0 to 2 with a total score of 7–10 as normal and the highest score of 10 representing the optimal condition (1, 2). The Apgar score has been used worldwide as a vitality index for almost every newborn immediately after birth (1). Although its use has been criticized due to the problems of accuracy, reproducibility, and universality, the American Academy of Pediatrics and the American College of Obstetrics and Gynecology endorsed its continued use in their 2015 statement (3) because of its abilities to assess the need for and response to resuscitation and robust associations with the risks of infant morbidity and mortality (4, 5).

A low Apgar score represents combined effects of various pre- and perinatal risk factors, which may predict non-optimal brain development (6–10). There is mounting evidence that a low Apgar score, commonly defined as a score <7, has been linked with certain mental disorders in later life, such as Autism Spectrum Disorder (ASD), Obsessive-Compulsive Disorder (OCD), and Attention Deficit Hyperactivity Disorder (ADHD) (10–15). However, the evidence on the associations with other mental disorders is lacking. More importantly, most prior studies considered an Apgar score of 7–9 to be “normal” and did not investigate whether the scores of 7–9 would be associated with a higher risk of mental disorders. Quantifying such associations is important as suggested by recent findings that even the scores of 7–9 were associated with a higher risk of adverse short- and long-term outcomes, such as neonatal mortality, morbidity, cerebral palsy, and epilepsy in childhood (16, 17).

In this population-based study, we hypothesized that the risk of mental disorders would increase with decreasing Apgar scores, even within the ‘normal' range 7–9. Taking advantage of a Danish register-based cohort with up to 38 years of follow-up, we investigated the associations between 5-min Apgar scores and the risks of a full spectrum of mental disorders using population-based analyses.

## Methods

### Study Population

We conducted a population-based cohort study using data from Danish national registers (18). A total of 2,272,473 live singletons were identified during 1978–2015 from the Danish Medical Birth Registry (19). We excluded 1,001 births with missing information on sex and 41,252 births with no valid information on 5-min Apgar scores (including the score of 0) (11). We further excluded 16,398 infants who died or emigrated from Denmark before the age of 1 year. The final cohort comprised 2,213,822 births.

### Main Exposures

Apgar scores at 5 min were the main exposures. We categorized them into three groups of compromised scores (1–3, 4–6, 7–9) and one optimal score (10). In addition, we analyzed Apgar scores in the way of each score (1, 2, 3, 4, 5, 6, 7, 8, 9, and 10).

### Outcomes of Interest

Information on mental disorders was obtained from the Danish Psychiatric Central Research Register (PCRR) and the National Patient Register (NPR) (20, 21). The PCRR contains all admissions to psychiatric inpatient facilities since 1969 and contacts to outpatient psychiatric departments and emergency care units since 1995. The NPR includes hospital discharge diagnoses since 1977 and outpatient and emergency diagnoses since 1995. The diagnostic system used was the Danish modification of the International Classification of Diseases, Eighth Revision (ICD-8) from 1969 to 1993 and Tenth Revision (ICD-10) from 1994 onwards. Details of the specific diagnoses included in each group of disorders are presented in Supplementary Table S1 (22–24). For each mental disorder, the date of onset was defined as the first day of the first contact. Individuals with more than one disorder were included in the numerator for each specific disorder.

### Potential Confounders

We included the following potential confounders based on causal diagrams using directed acyclic graphs (Supplementary Figure S1): sex (male/female), calendar period of birth (1978–1985/1986–1994/1995–2005/2006–2015), gestational age at childbirth (≤ 31/32–33/34–36/37–38/39–40/41/≥42 weeks), birth weight percentiles (<10th/10th−90th/>90th centile) using the distribution of sex and year of delivery of the entire study population as the standard, parental psychiatric history at delivery (yes/no), and other maternal characteristics (parity [1/2/≥3 children], age at birth [ <20/20–24/25–29/30–34/ ≥35 years], smoking during pregnancy [yes/no], highest attained level of education [0–9/10–14/≥15 years], cohabitation with a partner [yes/no], residence [Copenhagen/cities with 1,00,000 or more inhabitants/other], birth country [Denmark/others]). Missing data for each variable were coded using a missing data indicator.

### Statistical Analysis

All included children were followed up from the earliest possible age at onset of the disorder (for each disorder separately) until the date of the first diagnosis, death, emigration, or December 31, 2016, whichever came first. Data were analyzed from December 2019 through June 2020. Cox regression was used to estimate hazard ratios (HRs) with a 95% CI to assess the associations between Apgar score and mental disorders, with participant's age as the time scale. Because there was evidence of non-proportional hazards, we split person time by attained age such that associations were allowed to vary over time. Age groups were set with childhood (≤ 18 years) and early adulthood (19–39 years). Regarding the analyses for early adulthood, we excluded some categories of mental disorders not commonly diagnosed in adulthood, such as autism.

In sensitivity analyses, we investigated potential sex specificity of risks of mental disorders by repeating the analyses in male or female individuals. We restricted the analyses to individuals without congenital malformations of the nervous system and chromosomal abnormalities to preclude the potential consequences of those defects on mental disorders and Apgar score. Owing to ICD code changes in 1994 and integration of registered data from three departments (inpatient, outpatient, and emergency) in 1995, we performed sub-analyses restricted to individuals born after 1994. In addition, we stratified the participants by gestational age at birth or birth weight percentile to assess whether the risk pattern was modified by fetal maturity or growth status *in utero*. All analyses were conducted using SAS 9.4 (SAS Institute, Cary, NC).

### Ethical Approval

The study was approved by the Data Protection Agency (No. 2013-41-2569). By Danish law, no informed consent is required for a registry-based study using anonymized data.

## Results

Of the 2,213,822 singleton live newborn infants, 1,47,984 (6.68%) were assigned compromised 5-min Apgar score (scores of 1–3:0.09%, 4–6:0.49%, 7–9: 6.10%). Compared with those with a score of 10, infants with compromised scores are more likely to be male and to have a parental history of mental disorders. Mothers of newborns with compromised scores are more likely to be primiparous, to bear a child before age 20 or after 35, to smoke during pregnancy, to live alone, and to have a low education level (Table 1).

**Table 1:** Baseline characteristics according to Apgar score at 5 min, live singleton births in Denmark from 1978 to 2015.

**Characteristics**	**Apgar 1–3 (n = 2083)**	**Apgar 4–6 (n = 10833)**	**Apgar 7–9 (*n* = 135,068)**	**Apgar 10 (*n* = 2,065,838)**
**Sex**				
Male	1,149 (55.2)	6,168 (56.9)	76,258 (56.5)	1,052,202 (50.9)
Female	934 (44.8)	4,665 (43.1)	58,810 (43.5)	1,013,636 (49.1)
**Calender year of birth**				
1978–1985	291 (14.0)	1,944 (18.0)	20,592 (15.3)	398,633 (19.3)
1986–1994	477 (22.9)	2,656 (24.5)	31,622 (23.4)	504,430 (24.4)
1995–2005	611 (29.3)	3,453 (31.9)	45,530 (33.7)	632,435 (30.6)
2006–2015	704 (33.8)	2,780 (25.7)	37,324 (27.6)	530,340 (25.7)
**Maternal parity**				
1	1,139 (54.7)	6,258 (57.8)	73,061 (54.1)	911,448 (44.1)
2	612 (29.4)	3,019 (27.9)	41,458 (30.7)	778,448 (37.7)
≥3	332 (15.9)	1,556 (14.4)	20,549 (15.2)	375,942 (18.2)
**Maternal age at birth (years)**				
<20	35 (1.7)	252 (2.3)	2,499 (1.9)	36,413 (1.8)
20–24	349 (16.8)	1,881 (17.4)	22,110 (16.4)	337,310 (16.3)
25–29	683 (32.8)	3,803 (35.1)	48,320 (35.8)	739,226 (35.8)
30–34	677 (32.5)	3,156 (29.1)	41,258 (30.6)	647,121 (31.3)
≥35	339 (16.3)	1,741 (16.1)	20,881 (15.5)	305,768 (14.8)
**Maternal smoking during pregnancy^a^**				
Yes	316 (20.4)	1,576 (21.0)	18,505 (18.9)	273,737 (19.5)
No	1,152 (74.2)	5,547 (73.9)	75,597 (77.0)	1,078,105 (76.9)
Missing	84 (5.4)	386 (5.1)	4,022 (4.1)	49,771 (3.6)
**Maternal education at childbirth (years)**				
0–9	600 (28.8)	3,205 (29.6)	35661 (26.4)	556962 (27.0)
10–14	893 (42.9)	4,766 (44.0)	60,357 (44.7)	892,458 (43.2)
≥15	539 (25.9)	2,649 (24.5)	36,875 (27.3)	579,258 (28.0)
Missing	51 (2.5)	213 (2.0)	2,175 (1.6)	37,160 (1.8)
**Maternal cohabitation at childbirth**				
Yes	1,020 (49.0)	5,355 (49.4)	68,410 (50.7)	1,144,783 (55.4)
No	1,060 (50.9)	5,472 (50.5)	66,613 (49.3)	920,495 (44.6)
Missing	3 (0.1)	6 (0.1)	45 (0.0)	560 (0.0)
**Maternal residence at childbirth**				
Copenhagen	188 (9.0)	1,052 (9.7)	14,601 (10.8)	231,791 (11.2)
Big cities ≥100,000 inhabitants	238 (11.4)	1,354 (12.5)	19,238 (14.2)	261,971 (12.7)
Others	1,657 (79.6)	8,427 (77.8)	101,229 (75.0)	1,572,076 (76.1)
**Maternal country of birth**				
Denmark	1,846 (88.6)	9,672 (89.3)	121,930 (90.3)	1,836,244 (88.9)
Others	233 (11.2)	1,137 (10.5)	12,961 (9.6)	226,544 (11.0)
Missing	4 (0.2)	24 (0.2)	177 (0.1)	3,050 (0.2)
**Maternal history of mental disorders**				
Yes	192 (9.2)	944 (8.7)	11,134 (8.2)	140,696 (6.8)
No	1,891 (90.8)	9,889 (91.3)	123,934 (91.8)	1,925,142 (93.2)
**Paternal history of mental disorders**				
Yes	158 (7.6)	708 (6.5)	8,133 (6.0)	114,468 (5.5)
No	1,919 (92.1)	10,096 (93.2)	126,542 (93.7)	1,946,511 (94.2)
Missing	6 (0.3)	29 (0.3)	393 (0.3)	4,859 (0.2)

*Expressed as frequency (percentage). Percentages have been rounded and may not total to 100.*

a *Maternal smoking during pregnancy was available since 1991*.

**Figure 1:**
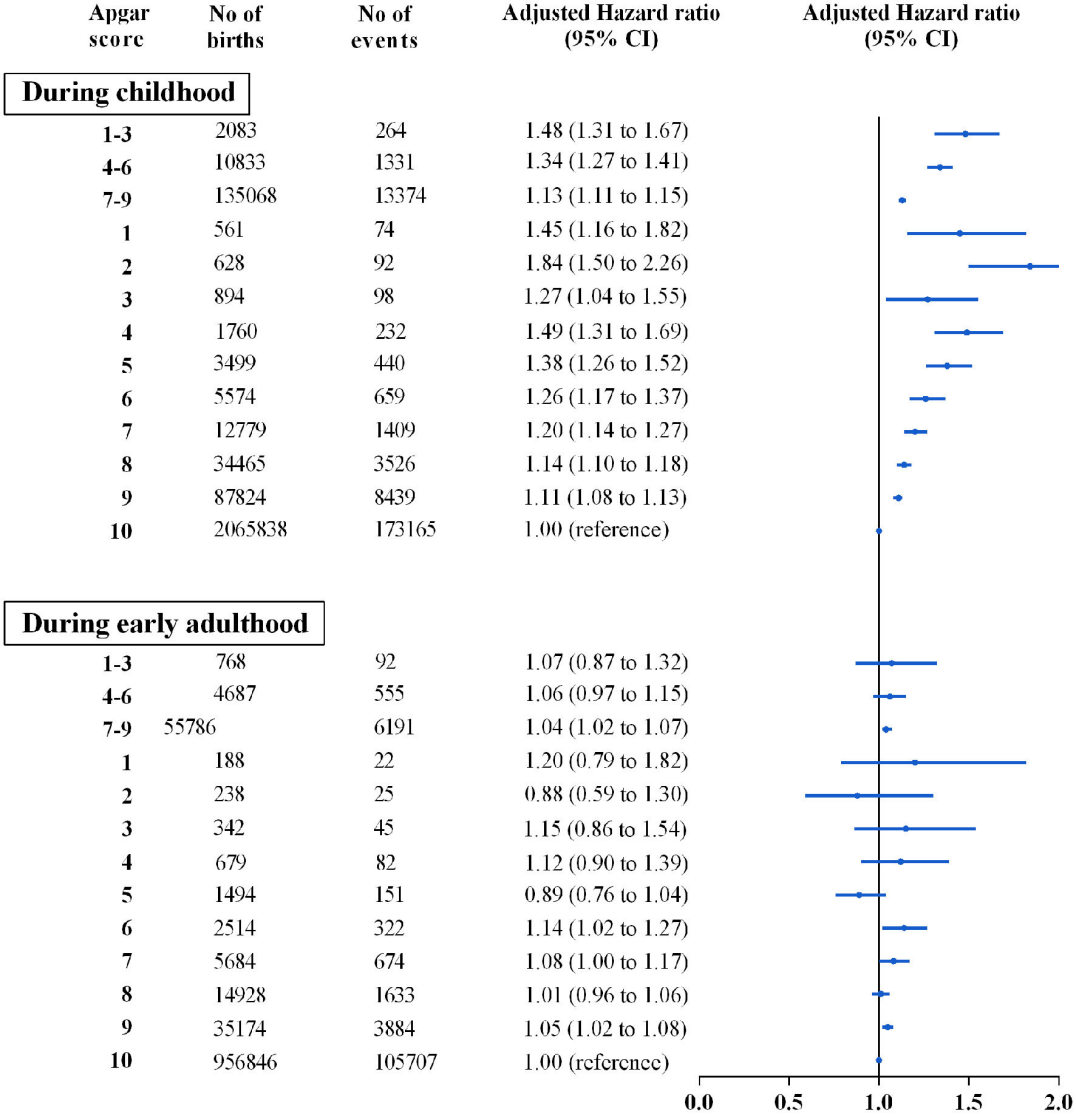
Associations between 5-min Apgar score and overall mental disorders by age periods during the first four decades of life. Hazard ratios are presented in the Cox regression models adjusted for parental psychiatric history, maternal characteristics (parity, age at birth, smoking during pregnancy, highest education level, cohabitation with a partner, residence, birth country), and birth characteristics (participant's sex, calendar year of birth, gestational age at birth and birth weight percentiles).

During up to 38 years of follow-up, 3,00,679 individuals received a diagnosis of mental disorders (356, 1886, 19 565, and 278 872 in groups of score at 1–3, 4–6, 7–9, and 10 respectively) (Figure 1). Throughout the childhood period, we found a dose-dependent pattern between compromised scores and the overall risks of mental disorders with HRs (95% CI) of 1.48 (1.31–1.67), 1.34 (1.27–1.41), and 1.13 (1.11–1.15) for scores of 1–3, 4–6, and 7–9, respectively, compared with the score of 10. Importantly, a dose-response association was also observed within the clinically “normal” score range of 7, 8, and 9: 1.20 (1.14–1.27), 1.14 (1.10–1.18), and 1.11 (1.08–1.13), respectively.

**Figure 2:**
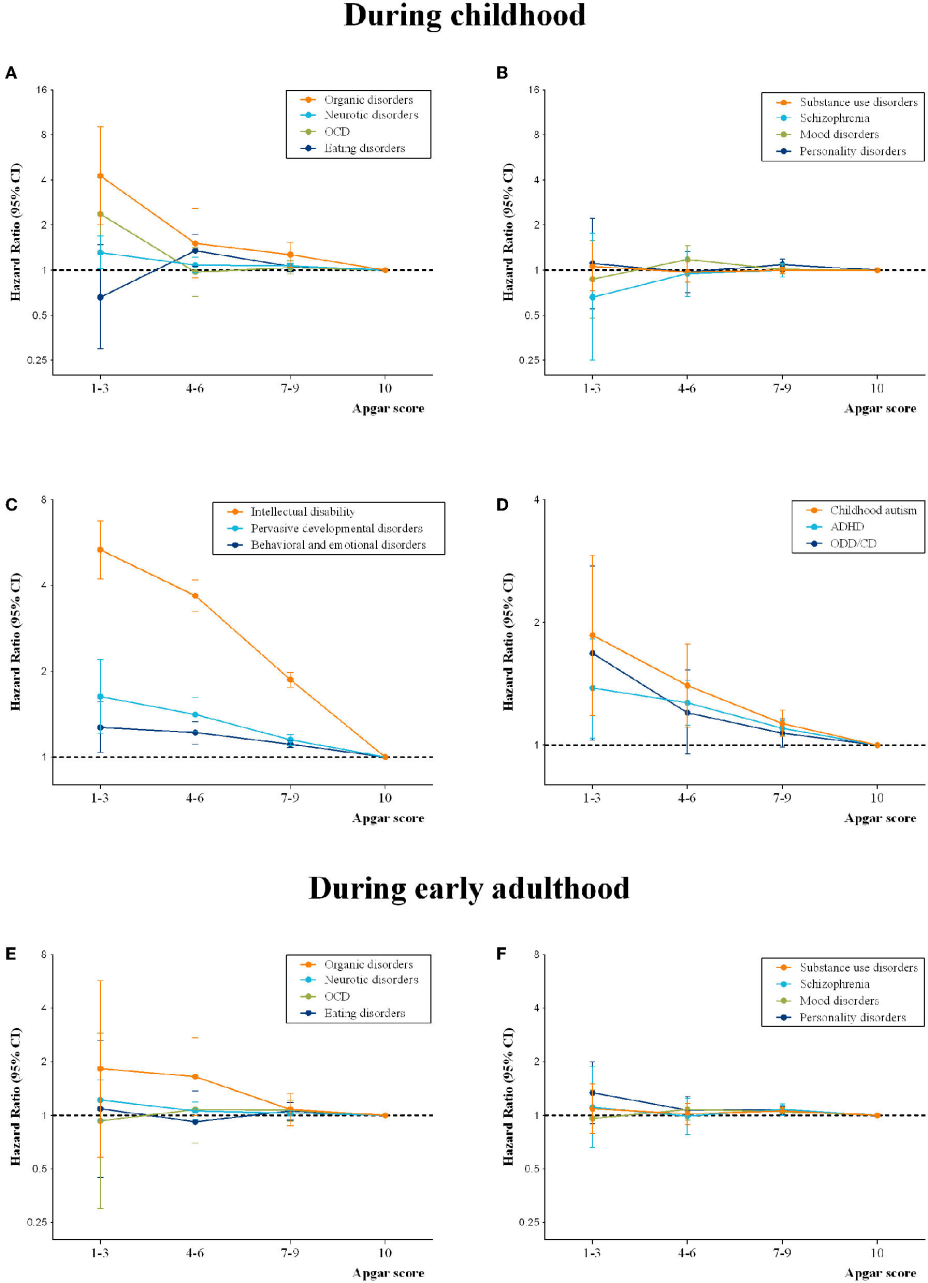
Associations between 5-min Apgar score and specific mental disorders during childhood **(A–D)** and early adulthood **(E,F)**. Hazard ratios are presented in the Cox regression models adjusted for parental psychiatric history, maternal characteristics (parity, age at birth, smoking during pregnancy, highest education level, cohabitation with a partner, residence, birth country), and birth characteristics (participant's sex, calendar year of birth, gestational age at birth and birth weight percentiles).

The relative risks of all studied subcategories of mental disorders in childhood were presented (Figure 2 and Supplementary Table S2). In the population analyses, dose-response gradients were observed across the entire score strata for intellectual disability (HR range: 1.87 to 5.33), organic disorders (HR range: 1.27 to 4.26), pervasive developmental disorders (HR range: 1.15 to 1.63), ADHD (HR range: 1.10 to 1.38), and for Oppositional Defiant Disorder/Conduct Disorder (ODD/CD) (HR range: 1.07 to 1.68), meanwhile, a higher risk (HR, 1.31) of neurotic disorders was seen in individuals with a score of 1–3. Of note, individuals with clinically “normal” score range of 7–9 have an 87% higher risk of intellectual disability (95% CI, 1.76–1.98) and 7~30% increased risks of other specific subcategories: organic disorders (1.27 [1.05–1.53]), neurotic disorders (1.07 [1.03–1.11]), pervasive developmental disorders (1.15 [1.10–1.20]), childhood autism (1.13 [1.05–1.22]), and ADHD (1.10 [1.06–1.15]).

During early adulthood, we did not find that compromised 5-min Apgar scores were associated with overall mental disorders (Figure 1). Nevertheless, higher risks but with low statistical precisions in the population cohort were found for some subtypes: schizophrenia (1.11 [0.66–1.88]), neurotic disorders (1.22 [0.94–1.58]), and personality disorders (1.34 [0.90–1.99]) in individuals with a score of 1–3, and organic disorders (1.65 [1.00–2.72]) in individuals with a score of 4–6 (Figure 2 andSupplementary Table S3).

In the sensitivity analysis, effect estimates from analyses stratified by gestational age at birth or birth weight percentile for gestational age were basically consistent with those observed in the main analysis (Supplementary Figures S2, S3). Stratified analyses by sex in the entire cohort showed no clear evidence of differences in associations between male and female individuals (Supplementary Tables S4, S5). Results from separate analyses restricted to individuals without diagnoses of congenital malformations of the nervous system or chromosomal abnormalities, or born after 1994, or without neonatal brain lesions were similar to those obtained in the primary analyses (Supplementary Tables S6–S9).

## Discussion

### Main Findings

In this large population-based cohort study, during childhood, we found individuals with even clinically “normal” Apgar score range of 7–9 still had higher risks of overall mental disorders and some specific diagnoses: organic disorders and a series of neurodevelopmental disorders (intellectual disability, pervasive developmental disorders, childhood autism, and ADHD). It is also interesting to observe that compromised Apgar scores were at elevated risks of developing organic disorders and neurotic disorders, which were reported for the first time. During early adulthood, compromised 5-min Apgar scores were not found to be associated with mental disorders.

### Comparisons With Other Studies

To our knowledge, this is the first study to examine the association of the full spectrum of mental disorders with the 5-min Apgar score. Our findings indicate the strongest associations for intellectual disability in childhood, which corroborates the results from previous studies (4, 15, 25, 26). Most of previous studies were based on the results of different non-standardized intelligence tests, and cross-sectional or descriptive designs (15, 25, 26), except a recent Swedish study, by virtue of clinically confirmed diagnosis and cohort design, reporting that term infants with low 5-min Apgar score had a higher risk of severe neurologic morbidity, including a 9-fold risk of intellectual disability (4). However, the Swedish study only captured cases before 14 years of age and only adjusted for year of birth, maternal age, parity, and smoking. Similarly, we observed 3~5-fold risks of intellectual disability in childhood (until 18 years of age), and we were able to adjust for not only the aforementioned confounders but also parental psychiatric history and socioeconomic status, indicating a more robust association. In addition, ADHD and autism were another two widely studied neurodevelopment disorders in relation to Apgar score during childhood, but the existing results were inconsistent (13, 27–31), which may be due to heterogeneity of methodology, in particular categorizations of Apgar scores and definition of outcomes. For example, some studies used pervasive developmental disorder (ICD-10 codes: F84) as a proxy to define autism (27, 28), which includes but is not limited to autism. To reduce the possibility of misclassification, we only focused on childhood autism–the typical and most severe type of autism–to explore the association. Our findings further support that compromised 5-min Apgar scores were associated with childhood autism.

Furthermore, we observed that individuals with a compromised 5-min Apgar score had higher risks of organic disorder and neurotic disorder during childhood, which have not been reported previously. These findings imply that less-than-optimal Apgar scores at birth may be an indicator for a broad scope of mental disorders in childhood, not merely neurodevelopmental disorders.

There have been scarce studies examining the association between low Apgar score and adulthood mental health. We did not find that compromised 5-min Apgar scores were associated with mental disorders during early adulthood, which may be attributed to the incomplete records of Apgar scores during the initial establishment of the Danish Medical Birth Register (MBR). We observed that participants with suboptimal Apgar scores at 5 min tended to have higher risks of organic disorders, schizophrenia, neurotic disorders, and personality disorders, and the low statistical precisions may probably be due to limited cases in the low Apgar score groups. Considering that the maximum attained age in our study was only up to 39 years, the follow-up between 19 and 39 years was not long enough to detect some late-onset mental disorders (e.g., dementia), therefore, future studies with extended follow-up to late adulthood are warranted.

Current guidelines recommend Apgar scores of 7 or higher to be reassuring, hence, infants with these scores are often assumed to constitute a homogeneous group. Nevertheless, recent studies showed that even reassuring Apgar scores of 7–9 are associated with higher risks of neonatal mortality, neonatal morbidity, and adverse long-term neurological outcomes, compared with an Apgar score of 10 (16, 17). We found a dose-response increasing the overall risk of mental disorders with decreasing Apgar score of 9 toward 7. Furthermore, individuals with “normal” scores of 7–9 carried increased risks of a wide range of neurodevelopmental disorders, such as intellectual disability, pervasive developmental disorders, childhood autism, and ADHD. Similarly, prior studies based on developmental screening scales found children aged 5 years with 5-min Apgar scores of 7–9 were more vulnerable on the emotional or physical health domain of the Early Development Instrument (14, 32). Recently, a large transnational study also suggested that low Apgar scores of 7–9 were associated with a higher risk of autistic disorder but without controlling for socioeconomic status and paternal psychiatric history (11). Our findings are in line with those of previous studies by showing that reassuring Apgar scores 7–9 are associated with various neurodevelopmental disorders in childhood. These findings support that 5-min Apgar scores routinely available in contemporary neonatal settings, even within the normal range 7–9, are not totally reassuring.

The causes of mental disorders are multifactorial. Adverse prenatal events (e.g., gestational diabetes mellitus, preterm, and restricted fetal growth) are important risk factors and could have a programming effect on fetal brain development, resulting in increased risk for psychopathology later in life (24, 33, 34). In this study, adjusting for gestational age at birth and fetal growth status did not substantially change the risks, indicating that preterm birth or restricted fetal growth do not strongly modify the relations between low Apgar scores at birth and subsequent mental disorders. Although Apgar scores are not clear on any causal pathway of pathogenesis, less-than-optimal Apgar scores at birth may be a potential sign of the cumulative effect of those adverse prenatal events. Especially, the clinically reassuring but suboptimal score range 7–9 may indicate subtle but still detrimental intrauterine insults which will act negatively on fetal brain development. In clinical settings, a distressed infant will receive resuscitation well before the 5-min Apgar score is assigned, so the score 7–9 could not well reflect severe conditions prior to the assessment (3). That may be one of the reasons we observed exposure to the scores 7–9 was associated with an increased risk of mental disorders. In this study, a novel finding that a compromised 5-min Apgar score was linked to increased risks of organic disorder and neurotic disorder was reported. As is known, organic disorder comprises a range of mental disorders based on a demonstrable etiology in cerebral disease, brain injury, or other insults leading to cerebral dysfunction (35). Increased risk of organic disorder with low Apgar score implies adverse prenatal insults (e.g., hypoxia-ischemia, white matter injury, reduced blood flow, malnutrition) exert a long-lasting impact on brain function in later life. With regard to neurotic disorder, its prevalence is relevant to low levels of socioeconomic status (SES) (36). In this study, we found individuals with compromised Apgar scores tended to be born in families with worse SES (e.g., mothers live alone and have a low education level). It is, therefore, possible that SES factors at least partially mediate the observed association between compromised Apgar scores and neurotic disorder. However, further elucidation of other potential mechanisms is needed, also whether Apgar scores can be used for predicting and screening at-risk infants needs further research, probably also in combination with other diagnostic tests.

### Strengths and Limitations

The study has several strengths. First, prospectively collected registry data including all live births in Denmark minimized the potential selection bias and recall bias. Second, a large sample size of over 2 million populations provided sufficient statistical power to perform detailed subgroup analyses that have not been studied previously. Last, the availability of rich sociodemographic and clinical information-enabled considerations of a wide range of important confounding factors.

Our study also has some limitations. First, we lacked information on interventions, which will be given to a distressed newborn before the first Apgar score is assigned and accordingly influence the Apgar score values (3). Whereas, failure to achieve an optimal score after resuscitation may signify some intrinsic defects and poor health status, hence, interventions during resuscitation are unlikely to affect the observed associations substantially. Also, we do not have the information on respiratory care (e.g., O_2_ cannula) and whether the patients were admitted to a sick baby room, which may be potential confounders. Second, on one hand, the Apgar score can be assessed subjectively without quality control and is prone to inter-observer variability. Whereas, the Apgar score has been shown to have good internal validity and could provide valid information on nation-level trends about newborn health (37). On the other hand, concerning large international variations in evaluating the Apgar score (37), the findings in this Danish cohort study are limited to generalize to other countries. Third, the study period spanned almost four decades, and advances in medical care over time and alterations in the diagnostic criteria may have influenced the exposures and outcomes. However, the inclusion of calendar time in the analyses would partially alleviate the effects of temporal changes in medical care. Last, the absence of information from primary care as well as the delayed inclusion of outpatient records in the PCRR and the NPR might result in an underestimation of the associations. This concern was partially relieved in our sensitivity analyses restricting to individuals born after 1994 when inpatient and outpatient records were integrated together, revealing similar results.

### Conclusions and Implications

Infants born with declining 5-min Apgar scores have incremental risks of a wide range of mental disorders, mainly during childhood but probably in adulthood, too. We found individuals with even clinically “normal” Apgar score range of 7–9 still had higher risks of overall mental disorders and some specific diagnoses: organic disorders and a series of neurodevelopmental disorders. Our findings suggested suboptimal Apgar score of 7–9 should be also considered as an alarming risk factor for subsequent mental disorders, which may help to identify and monitor at-risk neonates to minimize the risks of adverse psychiatric outcomes in their later life. The 5-min Apgar score should be taken into account in designing public health strategies that target populations at increased risks of specific mental disorders in both childhood and adulthood.

## Data Availability Statement

The raw data supporting the conclusions of this article will be made available by the authors, without undue reservation.

## Ethics Statement

The studies involving human participants were reviewed and approved by the Data Protection Agency (No. 2013-41-2569). By Danish law, no informed consent is required for a registry-based study using anonymized data.

## Author Contributions

JL had full access to all of the data in the study and took responsibility for the integrity of the data and the accuracy of the data analysis. FL conceptualized, designed, and reviewed the study. HH carried out the initial analyses, drafted the initial manuscript, and revised the manuscript. YY checked the statistical results and reviewed the manuscript. CO gave administrative, technical, or material support. All authors critically reviewed the manuscript for important intellectual content.

## Funding

This study was funded by the National Natural Science Foundation of China (Nos. 81761128035, 81930095, 82125032, and 82073570), the Novo Nordisk Foundation (NNF18OC0052029), the Danish Council for Independent Research (DFF-6110-00019B, 9039-00010B, and 1030-00012B), the Nordic Cancer Union (R275-A15770), and Karen Elise Jensens Fond (2016).

## Conflict of Interest

The authors declare that the research was conducted in the absence of any commercial or financial relationships that could be construed as a potential conflict of interest.

## Publisher's Note

All claims expressed in this article are solely those of the authors and do not necessarily represent those of their affiliated organizations, or those of the publisher, the editors and the reviewers. Any product that may be evaluated in this article, or claim that may be made by its manufacturer, is not guaranteed or endorsed by the publisher.

## Abbreviations

ASD: Autism Spectrum Disorder
OCD: Obsessive-Compulsive Disorder
ADHD: Attention Deficit Hyperactivity Disorder
PCRR: Psychiatric Central Research Register
NPR: National Patient Register
ICD: International Classification of Diseases
ODD/CD: Oppositional Defiant Disorder/Conduct Disorder
HR: Hazard Ratio
CI: confidence interval.
