# Supplementary material for: Five-Minute Apgar Score and the Risk of Mental Disorders During the First Four Decades of Life: A Nationwide Registry-Based Cohort Study in Denmark
Source: Front Med (Lausanne). 2022 Jan 14;8:796544. doi: 10.3389/fmed.2021.796544 (PMC8795588; doi:10.3389/fmed.2021.796544)
Supplement: Supplementary file 1 [file Table_1.DOCX]

**Table S1.** Diagnostic classification of mental disorders considered according to the ICD-10 and equivalent ICD-8 diagnoses.

| Diagnoses | Abbreviated name | ICD-8 | ICD-10 | Earliest possible age at onset (years) |
| --- | --- | --- | --- | --- |
| **Any mental disorder** | Any mental disorder | 290 – 315 | F00 – F99 | 1 |
| **Organic, including symptomatic, mental disorders** *Includes dementia in Alzheimer’s disease, vascular dementia, etc.* | Organic disorders | 290·09, 290·10, 290·11, 290·18, 290·19, 292·x9, 293·x9, 294·x9, 309·x9 | F00 – F09 | 1 |
| **Mental and behavioral disorders due to psychoactive substance use** *Includes use of alcohol, cannabis, cocaine, nicotine, opioids, sedatives, hypnotics, anxiolytics, etc.* | Substance use disorders | 291·x9, 294·39, 303·x9, 303·20, 303·28, 303·90, 304·x9 | F10 – F19 | 10 |
| **Schizophrenia and related disorders** *Includes schizophrenia, schizotypal disorders, schizoaffective disorders and other psychotic disorders.* | Schizophrenia | 295·x9, 296·89, 297·x9, 298·29-298·99, 299·04, 299·05, 299·09, 301·83 | F20 – F29 | 10 |
| **Mood disorders** *Includes bipolar disorder, depressive disorders, etc* | Mood disorders | 296·x9 (excluding 296·89), 298·09, 298·19, 300·49, 301·19 | F30 – F39 | 10 |
| **Neurotic, stress-related, and somatoform disorders** *Includes anxiety disorders, phobias, obsessive-compulsive disorders, etc.* | Neurotic disorders | 300·x9 (excluding 300·49), 305·x9, 305·68, 307·99 | F40 – F48 | 5 |
| **Obsessive-compulsive disorder** | Obsessive-compulsive disorder | 300.39 | F42 | 5 |
| **Eating disorders** *Includes anorexia nervosa, bulimia nervosa, etc.* | Eating disorders | 305·60, 306·50, 306·58, 306·59 | F50 | 1 |
| **Personality disorders** | Personality disorders | 301, 302 | F60–F69 | 10 |
| **Intellectual disability** | Intellectual disability | 311·xx, 312·xx, 313·xx, 314·xx, 315·xx | F70 – F79 | 1 |
| **Pervasive developmental disorders** *Includes autism spectrum disorder* | Developmental disorders | 299·00, 299·01, 299·02, 299·03 | F84 | 1 |
| **Childhood autism** | Childhood autism | 299.00 | F84.0 | 1 |
| **Behavioral and emotional disorders with onset usually occurring in childhood and adolescence** *Includes attention-deficit hyperactivity disorder, conduct disorders, childhood emotional disorders, etc* | Behavioral disorders | 306·x9, 308·0x | F90 – F98 | 1 |
| **Attention Deficit Hyperactivity Disorder** | ADHD | 308.01 | F90, F98.8 | 3 |
| **oppositional defiant disorder/conduct disorder** | ODD/CD | 308.03, 308.04 | F90.1, F91 | 3 |

ICD-10: International Classification of Diseases, 10th revision (1994-2016); ICD-8: Danish modification of the International Classification of Diseases, 8th revision (1978-1993)
