# Supplementary material for: Five-Minute Apgar Score and the Risk of Mental Disorders During the First Four Decades of Life: A Nationwide Registry-Based Cohort Study in Denmark
Source: Front Med (Lausanne). 2022 Jan 14;8:796544. doi: 10.3389/fmed.2021.796544 (PMC8795588; doi:10.3389/fmed.2021.796544)
Supplement: Supplementary file 2 [file Table_2.DOCX]

**Table S2.** Hazard ratios of specific mental disorders among individuals with compromised 5-minute Apgar scores compared to individuals with a score of 10 in childhood.

| **exposures and outcomes** | | **No of events** | **rate per 1000 person years** | **HR (95% CI), adjusted** ^a^ |
| --- | --- | --- | --- | --- |
| **Organic disorders** | |  |  |  |
| Apgar score 1~3 | | 7 | 0.29 | 4.26(2.01-9.01) |
| Apgar score 4~6 | | 14 | 0.10 | 1.51(0.89-2.58) |
| Apgar score 7~9 | | 123 | 0.07 | 1.27(1.05-1.53) |
| Apgar score 10 | | 1402 | 0.05 | 1.00 (ref) |
| **Substance use disorders** | |  |  |  |
| Apgar score 1~3 | | 28 | 2.72 | 1.06(0.73-1.54) |
| Apgar score 4~6 | | 152 | 2.47 | 0.97(0.83-1.14) |
| Apgar score 7~9 | | 1787 | 2.43 | 1.00(0.95-1.05) |
| Apgar score 10 | | 29430 | 2.49 | 1.00 (ref) |
| **Schizophrenia** | |  |  |  |
| Apgar score 1~3 | | <6 | 0.39 | NA |
| Apgar score 4~6 | | 32 | 0.52 | 0.95(0.67-1.34) |
| Apgar score 7~9 | | 387 | 0.52 | 1.00(0.90-1.11) |
| Apgar score 10 | | 5865 | 0.49 | 1.00 (ref) |
| **Mood disorders** | |  |  |  |
| Apgar score 1~3 | | 11 | 1.06 | 0.87(0.48-1.58) |
| Apgar score 4~6 | | 85 | 1.38 | 1.18(0.95-1.46) |
| Apgar score 7~9 | | 891 | 1.21 | 1.01(0.95-1.08) |
| Apgar score 10 | | 13935 | 1.18 | 1.00 (ref) |
| **Neurotic disorders** | |  |  |  |
| Apgar score 1~3 | | 60 | 3.34 | 1.31(1.02-1.69) |
| Apgar score 4~6 | | 278 | 2.64 | 1.08(0.96-1.22) |
| Apgar score 7~9 | | 3224 | 2.52 | 1.07(1.03-1.11) |
| Apgar score 10 | | 45568 | 2.25 | 1.00 (ref) |
|  | **OCD** |  |  |  |
|  | Apgar score 1~3 | 12 | 0.66 | 2.37(1.34-4.18) |
|  | Apgar score 4~6 | 28 | 0.26 | 0.97(0.67-1.41) |
|  | Apgar score 7~9 | 373 | 0.29 | 1.04(0.94-1.16) |
|  | Apgar score 10 | 5160 | 0.25 | 1.00 (ref) |
| **Eating disorders** | |  |  |  |
| Apgar score 1~3 | | 6 | 0.23 | 0.66(0.30-1.48) |
| Apgar score 4~6 | | 66 | 0.45 | 1.35(1.06-1.72) |
| Apgar score 7~9 | | 620 | 0.34 | 1.06(0.98-1.16) |
| Apgar score 10 | | 9157 | 0.32 | 1.00 (ref) |

HR=Hazard Ratio, CI=Confidential Interval, OCD= Obsessive-Compulsive Disorder

^a^ HRs in the population-based analysis were adjusted for parental psychiatric history, maternal characteristics (parity, age at birth, smoking during pregnancy, highest education level, cohabitation with a partner, residence, birth country) and birth characteristics (participant’s sex, calendar year of birth, gestational age at birth and birth weight percentiles).

**Table S2. (Continued)** Hazard ratios of specific mental disorders among individuals with compromised 5-minute Apgar scores compared to individuals with a score of 10 in childhood.

| **exposures and outcomes** | | **No of events** | **rate per 1000 person years** | **HR (95% CI), adjusted** ^a^ |
| --- | --- | --- | --- | --- |
| **Personality disorders** | |  |  |  |
| Apgar score 1~3 | | 8 | 0.77 | 1.11(0.55-2.22) |
| Apgar score 4~6 | | 40 | 0.65 | 0.97(0.71-1.33) |
| Apgar score 7~9 | | 507 | 0.69 | 1.09(0.99-1.19) |
| Apgar score 10 | | 7633 | 0.64 | 1.00 (ref) |
| **Intellectual disability** | |  |  |  |
| Apgar score 1~3 | | 72 | 2.86 | 5.33(4.21-6.74) |
| Apgar score 4~6 | | 256 | 1.77 | 3.68(3.24-4.18) |
| Apgar score 7~9 | | 1353 | 0.76 | 1.87(1.76-1.98) |
| Apgar score 10 | | 9579 | 0.34 | 1.00 (ref) |
| **Developmental disorders** | |  |  |  |
| Apgar score 1~3 | | 43 | 1.68 | 1.63(1.21-2.20) |
| Apgar score 4~6 | | 200 | 1.37 | 1.41(1.23-1.62) |
| Apgar score 7~9 | | 2011 | 1.12 | 1.15(1.10-1.20) |
| Apgar score 10 | | 22388 | 0.80 | 1.00 (ref) |
|  | **Childhood autism** |  |  |  |
|  | Apgar score 1~3 | 19 | 0.74 | 1.86(1.18-2.92) |
|  | Apgar score 4~6 | 72 | 0.49 | 1.40(1.11-1.77) |
|  | Apgar score 7~9 | 701 | 0.39 | 1.13(1.05-1.22) |
|  | Apgar score 10 | 7656 | 0.27 | 1.00 (ref) |
| **Behavioral disorders** | |  |  |  |
| Apgar score 1~3 | | 93 | 3.69 | 1.27(1.04-1.56) |
| Apgar score 4~6 | | 488 | 3.40 | 1.22(1.11-1.33) |
| Apgar score 7~9 | | 5313 | 3.01 | 1.11(1.08-1.14) |
| Apgar score 10 | | 65219 | 2.34 | 1.00 (ref) |
|  | **ADHD** |  |  |  |
|  | Apgar score 1~3 | 49 | 2.27 | 1.38(1.04-1.82) |
|  | Apgar score 4~6 | 249 | 2.00 | 1.27(1.12-1.44) |
|  | Apgar score 7~9 | 2621 | 1.72 | 1.10(1.06-1.15) |
|  | Apgar score 10 | 31202 | 1.30 | 1.00 (ref) |
|  | **ODD/CD** |  |  |  |
|  | Apgar score 1~3 | 16 | 0.74 | 1.68(1.03-2.75) |
|  | Apgar score 4~6 | 67 | 0.53 | 1.20(0.95-1.53) |
|  | Apgar score 7~9 | 692 | 0.45 | 1.07(0.99-1.16) |
|  | Apgar score 10 | 9034 | 0.37 | 1.00 (ref) |

HR=Hazard Ratio, CI=Confidential Interval, ADHD=Attention Deficit/Hyperactivity Disorder, ODD/CD=oppositional defiant disorder/conduct disorder

^a^ HRs in the population-based analysis were adjusted for parental psychiatric history, maternal characteristics (parity, age at birth, smoking during pregnancy, highest education level, cohabitation with a partner, residence, birth country) and birth characteristics (participant’s sex, calendar year of birth, gestational age at birth and birth weight percentiles).
