# Supplementary material for: Five-Minute Apgar Score and the Risk of Mental Disorders During the First Four Decades of Life: A Nationwide Registry-Based Cohort Study in Denmark
Source: Front Med (Lausanne). 2022 Jan 14;8:796544. doi: 10.3389/fmed.2021.796544 (PMC8795588; doi:10.3389/fmed.2021.796544)
Supplement: Supplementary file 3 [file Table_3.DOCX]

**Table S3.** Hazard ratios of specific mental disorders among individuals with compromised 5-minute Apgar scores compared to individuals with a score of 10 in early adulthood.

| **exposures and outcomes** | | **No of events** | **rate per 1000 person years** | **HR (95% CI), adjusted** ^a^ |
| --- | --- | --- | --- | --- |
| **Organic disorders** | |  |  |  |
| Apgar score 1~3 | | <6 | 0.79 | NA |
| Apgar score 4~6 | | 32 | 0.80 | 1.65(1.00-2.72) |
| Apgar score 7~9 | | 180 | 0.39 | 1.08(0.88-1.33) |
| Apgar score 10 | | 2361 | 0.27 | 1.00 (ref) |
| **Substance use disorders** | |  |  |  |
| Apgar score 1~3 | | 38 | 5.02 | 1.09(0.79-1.50) |
| Apgar score 4~6 | | 215 | 4.60 | 1.02(0.89-1.17) |
| Apgar score 7~9 | | 2459 | 4.67 | 1.06(1.02-1.11) |
| Apgar score 10 | | 39168 | 4.08 | 1.00 (ref) |
| **Schizophrenia** | |  |  |  |
| Apgar score 1~3 | | 14 | 1.77 | 1.11(0.66-1.88) |
| Apgar score 4~6 | | 73 | 1.49 | 0.99(0.78-1.25) |
| Apgar score 7~9 | | 851 | 1.55 | 1.08(1.00-1.16) |
| Apgar score 10 | | 12910 | 1.29 | 1.00 (ref) |
| **Mood disorders** | |  |  |  |
| Apgar score 1~3 | | 28 | 3.61 | 0.96(0.66-1.39) |
| Apgar score 4~6 | | 190 | 3.94 | 1.08(0.94-1.25) |
| Apgar score 7~9 | | 2015 | 3.72 | 1.05(1.00-1.10) |
| Apgar score 10 | | 34218 | 3.48 | 1.00 (ref) |
| **Neurotic disorders** | |  |  |  |
| Apgar score 1~3 | | 58 | 7.79 | 1.22(0.94-1.58) |
| Apgar score 4~6 | | 302 | 6.50 | 1.06(0.95-1.19) |
| Apgar score 7~9 | | 3205 | 6.11 | 1.03(0.99-1.06) |
| Apgar score 10 | | 54472 | 5.69 | 1.00 (ref) |
|  | **OCD** |  |  |  |
|  | Apgar score 1~3 | <6 | 0.38 | NA |
|  | Apgar score 4~6 | 21 | 0.42 | 1.08(0.70-1.66) |
|  | Apgar score 7~9 | 237 | 0.43 | 1.07(0.93-1.22) |
|  | Apgar score 10 | 3871 | 0.38 | 1.00 (ref) |
| **Eating disorders** | |  |  |  |
| Apgar score 1~3 | | <6 | 0.63 | NA |
| Apgar score 4~6 | | 25 | 0.51 | 0.92(0.62-1.37) |
| Apgar score 7~9 | | 340 | 0.62 | 1.06(0.94-1.18) |
| Apgar score 10 | | 5989 | 0.60 | 1.00 (ref) |
| **Personality disorders** | |  |  |  |
| Apgar score 1~3 | | 25 | 3.20 | 1.34(0.90-1.99) |
| Apgar score 4~6 | | 119 | 2.45 | 1.07(0.89-1.28) |
| Apgar score 7~9 | | 1287 | 2.36 | 1.07(1.01-1.13) |
| Apgar score 10 | | 20851 | 2.10 | 1.00 (ref) |

HR=Hazard Ratio, CI=Confidential Interval, OCD= Obsessive-Compulsive Disorder

^a^ HRs in the population-based analysis were adjusted for parental psychiatric history, maternal characteristics (parity, age at birth, smoking during pregnancy, highest education level, cohabitation with a partner, residence, birth country) and birth characteristics (participant’s sex, calendar year of birth, gestational age at birth and birth weight percentiles).
