# Supplementary material for: Five-Minute Apgar Score and the Risk of Mental Disorders During the First Four Decades of Life: A Nationwide Registry-Based Cohort Study in Denmark
Source: Front Med (Lausanne). 2022 Jan 14;8:796544. doi: 10.3389/fmed.2021.796544 (PMC8795588; doi:10.3389/fmed.2021.796544)
Supplement: Supplementary file 4 [file Table_4.DOCX]

**Table S4.** Hazard ratios of overall/specific mental disorders among male and female individuals with compromised 5-minute Apgar scores compared to individuals with a score of 10 in childhood.

|  | | **male** | |  | **female** | |
| --- | --- | --- | --- | --- | --- | --- |
| **exposures and outcomes** | | **No of events (rate per 1000 person years)** | **HR (95% CI), adjusted** |  | **No of events (rate per 1000 person years)** | **HR (95% CI), adjusted** |
| **Any mental disorder** | |  |  |  |  |  |
| Apgar score 1~3 | | 171 (13.02) | 1.61(1.38-1.87) |  | 93 (8.42) | 1.28(1.04-1.57) |
| Apgar score 4~6 | | 823 (10.57) | 1.36(1.27-1.46) |  | 508 (8.38) | 1.29(1.18-1.41) |
| Apgar score 7~9 | | 8057 (8.40) | 1.13(1.11-1.16) |  | 5317 (6.93) | 1.11(1.08-1.14) |
| Apgar score 10 | | 93718 (6.77) | 1.00 (ref) |  | 79447 (5.88) | 1.00 (ref) |
| **Organic disorders** | |  |  |  |  |  |
| Apgar score 1~3 | | 6 (0.46) | 5.77(2.55-13.04) |  | <6 (0.09) | NA |
| Apgar score 4~6 | | 10 (0.13) | 1.69(0.90-3.18) |  | <6 (0.07) | NA |
| Apgar score 7~9 | | 82 (0.09) | 1.34(1.06-1.69) |  | 41 (0.05) | 1.15(0.83-1.60) |
| Apgar score 10 | | 837 (0.06) | 1.00 (ref) |  | 565 (0.04) | 1.00 (ref) |
| **Substance use disorders** | |  |  |  |  |  |
| Apgar score 1~3 | | 19 (3.38) | 1.23(0.78-1.93) |  | 9 (1.93) | 0.82(0.43-1.57) |
| Apgar score 4~6 | | 94 (2.69) | 0.99(0.81-1.22) |  | 58 (2.18) | 0.94(0.72-1.22) |
| Apgar score 7~9 | | 1065 (2.60) | 1.01(0.95-1.07) |  | 722 (2.22) | 0.99(0.92-1.07) |
| Apgar score 10 | | 16094 (2.67) | 1.00 (ref) |  | 13336 (2.30) | 1.00 (ref) |
| **Schizophrenia** | |  |  |  |  |  |
| Apgar score 1~3 | | <6 (0.53) | NA |  | <6 (0.21) | NA |
| Apgar score 4~6 | | 19 (0.54) | 1.11(0.70-1.74) |  | 13 (0.49) | 0.78(0.45-1.35) |
| Apgar score 7~9 | | 176 (0.43) | 0.92(0.79-1.08) |  | 211 (0.65) | 1.07(0.93-1.24) |
| Apgar score 10 | | 2658 (0.44) | 1.00 (ref) |  | 3207 (0.55) | 1.00 (ref) |
| **Mood disorders** | |  |  |  |  |  |
| Apgar score 1~3 | | <6 (0.89) | NA |  | 6 (1.28) | 0.69(0.31-1.54) |
| Apgar score 4~6 | | 27 (0.77) | 1.10(0.75-1.61) |  | 58 (2.18) | 1.22(0.94-1.58) |
| Apgar score 7~9 | | 295 (0.72) | 1.02(0.90-1.15) |  | 596 (1.83) | 1.01(0.93-1.10) |
| Apgar score 10 | | 3983 (0.66) | 1.00 (ref) |  | 9952 (1.72) | 1.00 (ref) |
| **Neurotic disorders** | |  |  |  |  |  |
| Apgar score 1~3 | | 27 (2.74) | 1.48(1.01-2.16) |  | 33 (4.06) | 1.19(0.84-1.67) |
| Apgar score 4~6 | | 127 (2.12) | 1.21(1.01-1.44) |  | 151 (3.33) | 1.00(0.85-1.17) |
| Apgar score 7~9 | | 1252 (1.75) | 1.04(0.98-1.10) |  | 1972 (3.5) | 1.09(1.04-1.14) |
| Apgar score 10 | | 15701 (1.52) | 1.00 (ref) |  | 29867 (3.01) | 1.00 (ref) |
|  | **OCD** |  |  |  |  |  |
|  | Apgar score 1~3 | 8 (0.81) | 3.41(1.70-6.86) |  | <6 (0.49) | NA |
|  | Apgar score 4~6 | 13 (0.22) | 0.92(0.53-1.59) |  | 15 (0.33) | 1.02(0.61-1.70) |
|  | Apgar score 7~9 | 177 (0.25) | 1.02(0.87-1.19) |  | 196 (0.34) | 1.07(0.92-1.23) |
|  | Apgar score 10 | 2262 (0.22) | 1.00 (ref) |  | 2898 (0.29) | 1.00 (ref) |
| **Eating disorders** | |  |  |  |  |  |
| Apgar score 1~3 | | <6 (0.35) | NA |  | <6 (0.09) | NA |
| Apgar score 4~6 | | 11 (0.13) | 1.32(0.72-2.40) |  | 55 (0.87) | 1.36(1.04-1.78) |
| Apgar score 7~9 | | 117 (0.12) | 1.28(1.06-1.56) |  | 503 (0.64) | 1.03(0.94-1.12) |
| Apgar score 10 | | 1099 (0.08) | 1.00 (ref) |  | 8058 (0.58) | 1.00 (ref) |

HR=Hazard Ratio, CI=Confidential Interval, OCD= Obsessive-Compulsive Disorder

Cox models were adjusted for parental psychiatric history, maternal characteristics (parity, age at birth, smoking during pregnancy, highest education level, cohabitation with a partner, residence, birth country) and birth characteristics (calendar year of birth, gestational age at birth and birth weight percentiles).

**Table S4. (Continued)** Hazard ratios of overall/specific mental disorders among male and female individuals with compromised 5-minute Apgar scores compared to individuals with a score of 10 in childhood.

|  | | **male** | |  | **female** | |
| --- | --- | --- | --- | --- | --- | --- |
| **exposures and outcomes** | | **No of events (rate per 1000 person years)** | **HR (95% CI), adjusted** |  | **No of events (rate per 1000 person years)** | **HR (95% CI), adjusted** |
| **Personality disorders** | |  |  |  |  |  |
| Apgar score 1~3 | | <6 (0.35) | NA |  | 6 (1.28) | 1.15(0.52-2.57) |
| Apgar score 4~6 | | 12 (0.34) | 0.98(0.55-1.74) |  | 28 (1.05) | 0.97(0.67-1.41) |
| Apgar score 7~9 | | 120 (0.29) | 0.93(0.77-1.12) |  | 387 (1.19) | 1.15(1.04-1.28) |
| Apgar score 10 | | 1860 (0.31) | 1.00 (ref) |  | 5773 (0.99) | 1.00 (ref) |
| **Intellectual disability** | |  |  |  |  |  |
| Apgar score 1~3 | | 46 (3.33) | 4.97(3.71-6.67) |  | 26 (2.29) | 6.08(4.12-8.98) |
| Apgar score 4~6 | | 164 (2.00) | 3.45(2.95-4.04) |  | 92 (1.47) | 4.17(3.37-5.15) |
| Apgar score 7~9 | | 887 (0.89) | 1.81(1.68-1.95) |  | 466 (0.59) | 1.98(1.79-2.19) |
| Apgar score 10 | | 6032 (0.42) | 1.00 (ref) |  | 3547 (0.26) | 1.00 (ref) |
| **Developmental disorders** | |  |  |  |  |  |
| Apgar score 1~3 | | 35 (2.50) | 1.73(1.24-2.41) |  | 8 (0.69) | 1.35(0.67-2.70) |
| Apgar score 4~6 | | 158 (1.91) | 1.42(1.21-1.66) |  | 42 (0.66) | 1.40(1.03-1.91) |
| Apgar score 7~9 | | 1576 (1.58) | 1.13(1.08-1.20) |  | 435 (0.55) | 1.19(1.08-1.31) |
| Apgar score 10 | | 16954 (1.19) | 1.00 (ref) |  | 5434 (0.39) | 1.00 (ref) |
|  | **Childhood autism** |  |  |  |  |  |
|  | Apgar score 1~3 | 18 (1.28) | 2.21(1.39-3.52) |  | <6 (0.09) | NA |
|  | Apgar score 4~6 | 56 (0.67) | 1.34(1.03-1.75) |  | 16 (0.25) | 1.68(1.02-2.76) |
|  | Apgar score 7~9 | 569 (0.57) | 1.12(1.02-1.22) |  | 132 (0.17) | 1.20(1.00-1.43) |
|  | Apgar score 10 | 6066 (0.42) | 1.00 (ref) |  | 1590 (0.11) | 1.00 (ref) |
| **Behavioral disorders** | |  |  |  |  |  |
| Apgar score 1~3 | | 67 (4.88) | 1.31(1.03-1.66) |  | 26 (2.27) | 1.17(0.80-1.72) |
| Apgar score 4~6 | | 345 (4.26) | 1.21(1.09-1.34) |  | 143 (2.29) | 1.25(1.06-1.47) |
| Apgar score 7~9 | | 3827 (3.89) | 1.13(1.09-1.17) |  | 1486 (1.90) | 1.06(1.01-1.12) |
| Apgar score 10 | | 43131 (3.06) | 1.00 (ref) |  | 22088 (1.61) | 1.00 (ref) |
|  | **ADHD** |  |  |  |  |  |
|  | Apgar score 1~3 | 42 (3.57) | 1.55(1.15-2.10) |  | 7 (0.71) | 0.80(0.38-1.68) |
|  | Apgar score 4~6 | 190 (2.70) | 1.26(1.09-1.45) |  | 59 (1.09) | 1.32(1.02-1.71) |
|  | Apgar score 7~9 | 2009 (2.37) | 1.11(1.06-1.16) |  | 612 (0.91) | 1.08(0.99-1.17) |
|  | Apgar score 10 | 22330 (1.83) | 1.00 (ref) |  | 8872 (0.75) | 1.00 (ref) |
|  | **ODD/CD** |  |  |  |  |  |
|  | Apgar score 1~3 | 13 (1.09) | 1.74(1.01-3.01) |  | <6 (0.30) | NA |
|  | Apgar score 4~6 | 54 (0.76) | 1.21(0.93-1.59) |  | 13 (0.24) | 1.19(0.69-2.06) |
|  | Apgar score 7~9 | 553 (0.65) | 1.07(0.98-1.17) |  | 139 (0.21) | 1.06(0.89-1.26) |
|  | Apgar score 10 | 6848 (0.56) | 1.00 (ref) |  | 2186 (0.18) | 1.00 (ref) |

HR=Hazard Ratio, CI=Confidential Interval, ADHD=Attention Deficit Hyperactivity Disorder, ODD/CD=oppositional defiant disorder/conduct disorder

Cox models were adjusted for parental psychiatric history, maternal characteristics (parity, age at birth, smoking during pregnancy, highest education level, cohabitation with a partner, residence, birth country) and birth characteristics (calendar year of birth, gestational age at birth and birth weight percentiles).
