# Supplementary material for: Five-Minute Apgar Score and the Risk of Mental Disorders During the First Four Decades of Life: A Nationwide Registry-Based Cohort Study in Denmark
Source: Front Med (Lausanne). 2022 Jan 14;8:796544. doi: 10.3389/fmed.2021.796544 (PMC8795588; doi:10.3389/fmed.2021.796544)
Supplement: Supplementary file 5 [file Table_5.DOCX]

**Table S5.** Hazard ratios of overall/specific mental disorders among male and female individuals with compromised 5-minute Apgar scores compared to individuals with a score of 10 in early adulthood.

|  | | **male** | |  | **female** | |
| --- | --- | --- | --- | --- | --- | --- |
| **exposures and outcomes** | | **No of events (rate per 1000 person years)** | **HR (95% CI), adjusted** |  | **No of events (rate per 1000 person years)** | **HR (95% CI), adjusted** |
| **Any mental disorder** | |  |  |  |  |  |
| Apgar score 1~3 | | 43 (12.53) | 1.07(0.79-1.44) |  | 49 (17.08) | 1.07(0.81-1.42) |
| Apgar score 4~6 | | 266 (11.76) | 1.03(0.91-1.16) |  | 289 (16.70) | 1.08(0.96-1.21) |
| Apgar score 7~9 | | 2992 (11.71) | 1.05(1.01-1.09) |  | 3199 (15.52) | 1.03(1.00-1.07) |
| Apgar score 10 | | 46695 (10.54) | 1.00 (ref) |  | 59012 (14.18) | 1.00 (ref) |
| **Organic disorders** | |  |  |  |  |  |
| Apgar score 1~3 | | NA | NA |  | <6 (1.74) | NA |
| Apgar score 4~6 | | 20 (0.88) | 1.25(0.59-2.65) |  | 12 (0.69) | 2.23(1.13-4.37) |
| Apgar score 7~9 | | 93 (0.36) | 1.00(0.76-1.32) |  | 87 (0.42) | 1.19(0.88-1.61) |
| Apgar score 10 | | 1248 (0.28) | 1.00 (ref) |  | 1113 (0.27) | 1.00 (ref) |
| **Substance use disorders** | |  |  |  |  |  |
| Apgar score 1~3 | | 20 (4.89) | 0.93(0.60-1.45) |  | 18 (5.18) | 1.35(0.85-2.15) |
| Apgar score 4~6 | | 136 (5.24) | 1.03(0.87-1.22) |  | 79 (3.80) | 1.00(0.80-1.25) |
| Apgar score 7~9 | | 1475 (5.15) | 1.04(0.98-1.09) |  | 984 (4.09) | 1.11(1.04-1.19) |
| Apgar score 10 | | 22799 (4.7) | 1.00 (ref) |  | 16369 (3.45) | 1.00 (ref) |
| **Schizophrenia** | |  |  |  |  |  |
| Apgar score 1~3 | | 7 (1.61) | 0.87(0.42-1.84) |  | 7 (1.97) | 1.51(0.72-3.17) |
| Apgar score 4~6 | | 40 (1.46) | 0.85(0.63-1.17) |  | 33 (1.53) | 1.23(0.87-1.73) |
| Apgar score 7~9 | | 527 (1.75) | 1.07(0.98-1.17) |  | 324 (1.30) | 1.10(0.98-1.23) |
| Apgar score 10 | | 7626 (1.5) | 1.00 (ref) |  | 5284 (1.08) | 1.00 (ref) |
| **Mood disorders** | |  |  |  |  |  |
| Apgar score 1~3 | | 12 (2.79) | 1.07(0.61-1.89) |  | 16 (4.64) | 0.89(0.54-1.45) |
| Apgar score 4~6 | | 83 (3.05) | 1.23(0.99-1.53) |  | 107 (5.11) | 0.99(0.82-1.20) |
| Apgar score 7~9 | | 748 (2.5) | 1.04(0.97-1.12) |  | 1267 (5.24) | 1.05(0.99-1.11) |
| Apgar score 10 | | 11481 (2.27) | 1.00 (ref) |  | 22737 (4.76) | 1.00 (ref) |
| **Neurotic disorders** | |  |  |  |  |  |
| Apgar score 1~3 | | 33 (7.98) | 1.66(1.18-2.34) |  | 25 (7.55) | 0.91(0.61-1.35) |
| Apgar score 4~6 | | 128 (4.83) | 1.05(0.88-1.25) |  | 174 (8.73) | 1.07(0.92-1.25) |
| Apgar score 7~9 | | 1329 (4.54) | 1.00(0.95-1.06) |  | 1876 (8.08) | 1.05(1.00-1.10) |
| Apgar score 10 | | 21245 (4.28) | 1.00 (ref) |  | 33227 (7.2) | 1.00 (ref) |
|  | **OCD** |  |  |  |  |  |
|  | Apgar score 1~3 | NA | NA |  | <6 (0.84) | NA |
|  | Apgar score 4~6 | 8 (0.29) | 1.12(0.56-2.26) |  | 13 (0.6) | 1.06(0.61-1.83) |
|  | Apgar score 7~9 | 86 (0.28) | 1.07(0.86-1.34) |  | 151 (0.6) | 1.06(0.90-1.26) |
|  | Apgar score 10 | 1273 (0.25) | 1.00 (ref) |  | 2598 (0.53) | 1.00 (ref) |
| **Eating disorders** | |  |  |  |  |  |
| Apgar score 1~3 | | <6 (0.23) | NA |  | <6 (1.13) | NA |
| Apgar score 4~6 | | <6 (0.07) | NA |  | 23 (1.07) | 0.89(0.59-1.35) |
| Apgar score 7~9 | | 21 (0.07) | 1.40(0.89-2.20) |  | 319 (1.29) | 1.04(0.93-1.16) |
| Apgar score 10 | | 236 (0.05) | 1.00 (ref) |  | 5753 (1.18) | 1.00 (ref) |
| **Personality disorders** | |  |  |  |  |  |
| Apgar score 1~3 | | 8 (1.85) | 1.29(0.64-2.58) |  | 17 (4.88) | 1.37(0.85-2.20) |
| Apgar score 4~6 | | 41 (1.49) | 1.09(0.80-1.49) |  | 78 (3.68) | 1.06(0.85-1.33) |
| Apgar score 7~9 | | 434 (1.44) | 1.10(1.00-1.21) |  | 853 (3.49) | 1.06(0.98-1.13) |
| Apgar score 10 | | 6278 (1.23) | 1.00 (ref) |  | 14573 (3.02) | 1.00 (ref) |

HR=Hazard Ratio, CI=Confidential Interval, OCD= Obsessive-Compulsive Disorder

Cox models were adjusted for parental psychiatric history, maternal characteristics (parity, age at birth, smoking during pregnancy, highest education level, cohabitation with a partner, residence, birth country) and birth characteristics (calendar year of birth, gestational age at birth and birth weight percentiles).
