# Supplementary material for: Five-Minute Apgar Score and the Risk of Mental Disorders During the First Four Decades of Life: A Nationwide Registry-Based Cohort Study in Denmark
Source: Front Med (Lausanne). 2022 Jan 14;8:796544. doi: 10.3389/fmed.2021.796544 (PMC8795588; doi:10.3389/fmed.2021.796544)
Supplement: Supplementary file 6 [file Table_6.DOCX]

**Table S6.** Hazard ratios of overall/specific mental disorders among individuals without congenital malformations of the nervous system and chromosomal abnormalities born with compromised 5-minute Apgar scores VS a score of 10 in childhood.

| **exposures and outcomes** | | **No of events** | **rate per 1000 person years** | **HR (95% CI), adjusted** |
| --- | --- | --- | --- | --- |
| **Any mental disorder** | |  |  |  |
| Apgar score 1~3 | | 248 | 10.49 | 1.44(1.27-1.64) |
| Apgar score 4~6 | | 1258 | 9.27 | 1.31(1.24-1.38) |
| Apgar score 7~9 | | 12888 | 7.55 | 1.11(1.09-1.13) |
| Apgar score 10 | | 170543 | 6.26 | 1.00 (ref) |
| **Organic disorders** | |  |  |  |
| Apgar score 1~3 | | 7 | 0.30 | 4.49(2.12-9.52) |
| Apgar score 4~6 | | 14 | 0.10 | 1.60(0.94-2.73) |
| Apgar score 7~9 | | 113 | 0.07 | 1.22(1.00-1.48) |
| Apgar score 10 | | 1354 | 0.05 | 1.00 (ref) |
| **Substance use disorders** | |  |  |  |
| Apgar score 1~3 | | 28 | 2.80 | 1.09(0.75-1.58) |
| Apgar score 4~6 | | 149 | 2.48 | 0.97(0.83-1.14) |
| Apgar score 7~9 | | 1781 | 2.46 | 1.01(0.96-1.06) |
| Apgar score 10 | | 29324 | 2.50 | 1.00 (ref) |
| **Schizophrenia** | |  |  |  |
| Apgar score 1~3 | | <6 | 0.40 | NA |
| Apgar score 4~6 | | 32 | 0.53 | 0.97(0.68-1.38) |
| Apgar score 7~9 | | 384 | 0.53 | 1.01(0.91-1.12) |
| Apgar score 10 | | 5819 | 0.49 | 1.00 (ref) |
| **Mood disorders** | |  |  |  |
| Apgar score 1~3 | | 11 | 1.10 | 0.89(0.49-1.62) |
| Apgar score 4~6 | | 83 | 1.38 | 1.18(0.95-1.46) |
| Apgar score 7~9 | | 884 | 1.22 | 1.02(0.95-1.09) |
| Apgar score 10 | | 13871 | 1.18 | 1.00 (ref) |
| **Neurotic disorders** | |  |  |  |
| Apgar score 1~3 | | 59 | 3.38 | 1.33(1.03-1.71) |
| Apgar score 4~6 | | 271 | 2.64 | 1.09(0.96-1.22) |
| Apgar score 7~9 | | 3179 | 2.52 | 1.07(1.03-1.11) |
| Apgar score 10 | | 45245 | 2.24 | 1.00 (ref) |
|  | **OCD** |  |  |  |
|  | Apgar score 1~3 | 12 | 0.68 | 2.43(1.38-4.30) |
|  | Apgar score 4~6 | 28 | 0.27 | 0.99(0.68-1.44) |
|  | Apgar score 7~9 | 369 | 0.29 | 1.04(0.94-1.16) |
|  | Apgar score 10 | 5123 | 0.25 | 1.00 (ref) |
| **Eating disorders** | |  |  |  |
| Apgar score 1~3 | | 6 | 0.24 | 0.69(0.31-1.54) |
| Apgar score 4~6 | | 65 | 0.45 | 1.38(1.08-1.76) |
| Apgar score 7~9 | | 604 | 0.34 | 1.06(0.98-1.15) |
| Apgar score 10 | | 9053 | 0.32 | 1.00 (ref) |

HR=Hazard Ratio, CI=Confidential Interval, OCD= Obsessive-Compulsive Disorder

Cox models were adjusted for parental psychiatric history, maternal characteristics (parity, age at birth, smoking during pregnancy, highest education level, cohabitation with a partner, residence, birth country) and birth characteristics (participant’s sex, calendar year of birth, gestational age at birth and birth weight percentiles).

**Table S6. (Continued)** Hazard ratios of overall/specific mental disorders among individuals without congenital malformations of the nervous system and chromosomal abnormalities born with compromised 5-minute Apgar scores VS a score of 10 in childhood.

| **exposures and outcomes** | | **No of events** | **rate per 1000 person years** | **HR (95% CI), adjusted** |
| --- | --- | --- | --- | --- |
| **Personality disorders** | |  |  |  |
| Apgar score 1~3 | | 8 | 0.80 | 1.14(0.57-2.27) |
| Apgar score 4~6 | | 40 | 0.66 | 1.00(0.73-1.37) |
| Apgar score 7~9 | | 502 | 0.69 | 1.09(1.00-1.20) |
| Apgar score 10 | | 7587 | 0.64 | 1.00 (ref) |
| **Intellectual disability** | |  |  |  |
| Apgar score 1~3 | | 56 | 2.27 | 5.00(3.83-6.52) |
| Apgar score 4~6 | | 202 | 1.43 | 3.50(3.03-4.03) |
| Apgar score 7~9 | | 1033 | 0.58 | 1.69(1.58-1.80) |
| Apgar score 10 | | 8227 | 0.29 | 1.00 (ref) |
| **Developmental disorders** | |  |  |  |
| Apgar score 1~3 | | 39 | 1.57 | 1.55(1.13-2.12) |
| Apgar score 4~6 | | 193 | 1.36 | 1.42(1.23-1.64) |
| Apgar score 7~9 | | 1917 | 1.08 | 1.13(1.08-1.18) |
| Apgar score 10 | | 21856 | 0.78 | 1.00 (ref) |
|  | **Childhood autism** |  |  |  |
|  | Apgar score 1~3 | 16 | 0.64 | 1.65(1.01-2.69) |
|  | Apgar score 4~6 | 68 | 0.48 | 1.40(1.10-1.78) |
|  | Apgar score 7~9 | 657 | 0.37 | 1.11(1.02-1.20) |
|  | Apgar score 10 | 7384 | 0.26 | 1.00 (ref) |
| **Behavioral disorders** | |  |  |  |
| Apgar score 1~3 | | 91 | 3.72 | 1.29(1.05-1.58) |
| Apgar score 4~6 | | 474 | 3.38 | 1.22(1.11-1.34) |
| Apgar score 7~9 | | 5197 | 2.98 | 1.11(1.08-1.14) |
| Apgar score 10 | | 64495 | 2.33 | 1.00 (ref) |
|  | **ADHD** |  |  |  |
|  | Apgar score 1~3 | 48 | 2.29 | 1.39(1.05-1.85) |
|  | Apgar score 4~6 | 243 | 2.00 | 1.28(1.13-1.45) |
|  | Apgar score 7~9 | 2566 | 1.71 | 1.10(1.06-1.15) |
|  | Apgar score 10 | 30854 | 1.29 | 1.00 (ref) |
|  | **ODD/CD** |  |  |  |
|  | Apgar score 1~3 | 16 | 0.76 | 1.74(1.06-2.84) |
|  | Apgar score 4~6 | 63 | 0.51 | 1.17(0.91-1.50) |
|  | Apgar score 7~9 | 680 | 0.45 | 1.07(0.99-1.16) |
|  | Apgar score 10 | 8931 | 0.37 | 1.00 (ref) |

HR=Hazard Ratio, CI=Confidential Interval, ADHD=Attention Deficit Hyperactivity Disorder, ODD/CD=oppositional defiant disorder/conduct disorder

Cox models were adjusted for parental psychiatric history, maternal characteristics (parity, age at birth, smoking during pregnancy, highest education level, cohabitation with a partner, residence, birth country) and birth characteristics (participant’s sex, calendar year of birth, gestational age at birth and birth weight percentiles).
