# Supplementary material for: Five-Minute Apgar Score and the Risk of Mental Disorders During the First Four Decades of Life: A Nationwide Registry-Based Cohort Study in Denmark
Source: Front Med (Lausanne). 2022 Jan 14;8:796544. doi: 10.3389/fmed.2021.796544 (PMC8795588; doi:10.3389/fmed.2021.796544)
Supplement: Supplementary file 7 [file Table_7.DOCX]

**Table S7.** Hazard ratios of overall/specific mental disorders among individuals without congenital malformations of the nervous system and chromosomal abnormalities born with compromised 5-minute Apgar scores VS a score of 10 in early adulthood.

| **exposures and outcomes** | | **No of events** | **rate per 1000 person years** | **HR (95% CI), adjusted** |
| --- | --- | --- | --- | --- |
| **Any mental disorder** | |  |  |  |
| Apgar score 1~3 | | 91 | 14.79 | 1.08(0.88-1.33) |
| Apgar score 4~6 | | 544 | 13.94 | 1.06(0.97-1.15) |
| Apgar score 7~9 | | 6079 | 13.33 | 1.04(1.01-1.06) |
| Apgar score 10 | | 105082 | 12.29 | 1.00 (ref) |
| **Organic disorders** | |  |  |  |
| Apgar score 1~3 | | <6 | 0.81 | NA |
| Apgar score 4~6 | | 30 | 0.77 | 1.67(1.00-2.81) |
| Apgar score 7~9 | | 164 | 0.36 | 1.04(0.85-1.29) |
| Apgar score 10 | | 2300 | 0.27 | 1.00 (ref) |
| **Substance use disorders** | |  |  |  |
| Apgar score 1~3 | | 37 | 5.06 | 1.10(0.80-1.52) |
| Apgar score 4~6 | | 213 | 4.67 | 1.04(0.90-1.19) |
| Apgar score 7~9 | | 2428 | 4.68 | 1.07(1.02-1.11) |
| Apgar score 10 | | 38956 | 4.09 | 1.00 (ref) |
| **Schizophrenia** | |  |  |  |
| Apgar score 1~3 | | 14 | 1.83 | 1.16(0.68-1.96) |
| Apgar score 4~6 | | 71 | 1.49 | 0.99(0.78-1.25) |
| Apgar score 7~9 | | 836 | 1.54 | 1.08(1.00-1.16) |
| Apgar score 10 | | 12818 | 1.29 | 1.00 (ref) |
| **Mood disorders** | |  |  |  |
| Apgar score 1~3 | | 28 | 3.74 | 0.99(0.68-1.44) |
| Apgar score 4~6 | | 186 | 3.96 | 1.10(0.95-1.27) |
| Apgar score 7~9 | | 1983 | 3.72 | 1.05(1.00-1.10) |
| Apgar score 10 | | 34005 | 3.48 | 1.00 (ref) |
| **Neurotic disorders** | |  |  |  |
| Apgar score 1~3 | | 58 | 8.06 | 1.26(0.97-1.63) |
| Apgar score 4~6 | | 296 | 6.53 | 1.07(0.96-1.20) |
| Apgar score 7~9 | | 3140 | 6.07 | 1.02(0.99-1.06) |
| Apgar score 10 | | 54110 | 5.68 | 1.00 (ref) |
|  | **OCD** |  |  |  |
|  | Apgar score 1~3 | <6 | 0.39 | NA |
|  | Apgar score 4~6 | 21 | 0.44 | 1.12(0.72-1.72) |
|  | Apgar score 7~9 | 232 | 0.42 | 1.06(0.93-1.22) |
|  | Apgar score 10 | 3852 | 0.38 | 1.00 (ref) |
| **Eating disorders** | |  |  |  |
| Apgar score 1~3 | | <6 | 0.65 | NA |
| Apgar score 4~6 | | 25 | 0.52 | 0.95(0.64-1.41) |
| Apgar score 7~9 | | 335 | 0.62 | 1.06(0.94-1.18) |
| Apgar score 10 | | 5957 | 0.60 | 1.00 (ref) |
| **Personality disorders** | |  |  |  |
| Apgar score 1~3 | | 25 | 3.31 | 1.37(0.92-2.03) |
| Apgar score 4~6 | | 117 | 2.47 | 1.08(0.90-1.30) |
| Apgar score 7~9 | | 1271 | 2.37 | 1.07(1.01-1.13) |
| Apgar score 10 | | 20733 | 2.10 | 1.00 (ref) |

HR=Hazard Ratio, CI=Confidential Interval, OCD= Obsessive-Compulsive Disorder

Cox models were adjusted for parental psychiatric history, maternal characteristics (parity, age at birth, smoking during pregnancy, highest education level, cohabitation with a partner, residence, birth country) and birth characteristics (participant’s sex, calendar year of birth, gestational age at birth and birth weight percentiles).
