# Supplementary material for: Five-Minute Apgar Score and the Risk of Mental Disorders During the First Four Decades of Life: A Nationwide Registry-Based Cohort Study in Denmark
Source: Front Med (Lausanne). 2022 Jan 14;8:796544. doi: 10.3389/fmed.2021.796544 (PMC8795588; doi:10.3389/fmed.2021.796544)
Supplement: Supplementary file 8 [file Table_8.DOCX]

**Table S8.** Hazard ratios of overall/specific mental disorders among individuals in childhood born after 1994 born with compromised 5-minute Apgar scores VS a score of 10.

| **exposures and outcomes** | | **No of events** | **rate per 1000 person years** | **HR (95% CI), adjusted** |
| --- | --- | --- | --- | --- |
| **Any mental disorder** | |  |  |  |
| Apgar score 1~3 | | 148 | 12.97 | 1.53(1.30-1.79) |
| Apgar score 4~6 | | 706 | 11.47 | 1.31(1.22-1.42) |
| Apgar score 7~9 | | 7530 | 9.05 | 1.12(1.09-1.14) |
| Apgar score 10 | | 89289 | 7.63 | 1.00 (ref) |
| **Organic disorders** | |  |  |  |
| Apgar score 1~3 | | <6 | 0.18 | NA |
| Apgar score 4~6 | | <6 | 0.03 | NA |
| Apgar score 7~9 | | 39 | 0.05 | 1.45(1.04-2.02) |
| Apgar score 10 | | 367 | 0.03 | 1.00 (ref) |
| **Substance use disorders** | |  |  |  |
| Apgar score 1~3 | | 10 | 2.79 | 1.84(0.99-3.43) |
| Apgar score 4~6 | | 29 | 1.36 | 0.89(0.62-1.28) |
| Apgar score 7~9 | | 371 | 1.35 | 0.92(0.83-1.02) |
| Apgar score 10 | | 5921 | 1.55 | 1.00 (ref) |
| **Schizophrenia** | |  |  |  |
| Apgar score 1~3 | | <6 | 0.56 | NA |
| Apgar score 4~6 | | 13 | 0.61 | 0.96(0.56-1.66) |
| Apgar score 7~9 | | 161 | 0.59 | 1.01(0.86-1.18) |
| Apgar score 10 | | 2251 | 0.59 | 1.00 (ref) |
| **Mood disorders** | |  |  |  |
| Apgar score 1~3 | | <6 | 1.11 | NA |
| Apgar score 4~6 | | 42 | 1.98 | 1.31(0.96-1.77) |
| Apgar score 7~9 | | 431 | 1.57 | 1.06(0.96-1.17) |
| Apgar score 10 | | 5931 | 1.55 | 1.00 (ref) |
| **Neurotic disorders** | |  |  |  |
| Apgar score 1~3 | | 36 | 4.78 | 1.50(1.08-2.08) |
| Apgar score 4~6 | | 144 | 3.38 | 1.07(0.91-1.26) |
| Apgar score 7~9 | | 1783 | 3.17 | 1.08(1.03-1.13) |
| Apgar score 10 | | 22803 | 2.91 | 1.00 (ref) |
|  | **OCD** |  |  |  |
|  | Apgar score 1~3 | 8 | 1.05 | 2.58(1.29-5.19) |
|  | Apgar score 4~6 | 13 | 0.30 | 0.74(0.43-1.29) |
|  | Apgar score 7~9 | 214 | 0.38 | 0.96(0.83-1.10) |
|  | Apgar score 10 | 3021 | 0.38 | 1.00 (ref) |
| **Eating disorders** | |  |  |  |
| Apgar score 1~3 | | <6 | 0.33 | NA |
| Apgar score 4~6 | | 38 | 0.58 | 1.61(1.17-2.22) |
| Apgar score 7~9 | | 330 | 0.38 | 1.13(1.01-1.26) |
| Apgar score 10 | | 4182 | 0.34 | 1.00 (ref) |

HR=Hazard Ratio, CI=Confidential Interval, OCD= Obsessive-Compulsive Disorder

Cox models were adjusted for parental psychiatric history, maternal characteristics (parity, age at birth, smoking during pregnancy, highest education level, cohabitation with a partner, residence, birth country) and birth characteristics (participant’s sex, calendar year of birth, gestational age at birth and birth weight percentiles).

**Table S8. (Continued)** Hazard ratios of overall/specific mental disorders among individuals in childhood born after 1994 born with compromised 5-minute Apgar scores VS a score of 10.

| **exposures and outcomes** | | **No of events** | **rate per 1000 person years** | **HR (95% CI), adjusted** |
| --- | --- | --- | --- | --- |
| **Personality disorders** | |  |  |  |
| Apgar score 1~3 | | <6 | 1.12 | NA |
| Apgar score 4~6 | | 13 | 0.61 | 1.10(0.64-1.91) |
| Apgar score 7~9 | | 148 | 0.54 | 1.05(0.88-1.24) |
| Apgar score 10 | | 2180 | 0.57 | 1.00 (ref) |
| **Intellectual disability** | |  |  |  |
| Apgar score 1~3 | | 33 | 2.75 | 4.84(3.43-6.83) |
| Apgar score 4~6 | | 120 | 1.86 | 3.38(2.81-4.06) |
| Apgar score 7~9 | | 657 | 0.76 | 1.63(1.50-1.77) |
| Apgar score 10 | | 5039 | 0.42 | 1.00 (ref) |
| **Developmental disorders** | |  |  |  |
| Apgar score 1~3 | | 31 | 2.56 | 1.52(1.07-2.16) |
| Apgar score 4~6 | | 152 | 2.35 | 1.38(1.18-1.62) |
| Apgar score 7~9 | | 1577 | 1.83 | 1.13(1.07-1.19) |
| Apgar score 10 | | 17182 | 1.43 | 1.00 (ref) |
|  | **Childhood autism** |  |  |  |
|  | Apgar score 1~3 | 15 | 1.23 | 1.72(1.04-2.87) |
|  | Apgar score 4~6 | 62 | 0.95 | 1.41(1.09-1.81) |
|  | Apgar score 7~9 | 613 | 0.71 | 1.13(1.04-1.23) |
|  | Apgar score 10 | 6575 | 0.54 | 1.00 (ref) |
| **Behavioral disorders** | |  |  |  |
| Apgar score 1~3 | | 58 | 4.87 | 1.22(0.94-1.58) |
| Apgar score 4~6 | | 308 | 4.84 | 1.18(1.06-1.32) |
| Apgar score 7~9 | | 3558 | 4.18 | 1.10(1.06-1.14) |
| Apgar score 10 | | 41546 | 3.49 | 1.00 (ref) |
|  | **ADHD** |  |  |  |
|  | Apgar score 1~3 | 33 | 3.43 | 1.24(0.88-1.75) |
|  | Apgar score 4~6 | 184 | 3.49 | 1.24(1.07-1.44) |
|  | Apgar score 7~9 | 1999 | 2.85 | 1.08(1.04-1.14) |
|  | Apgar score 10 | 23396 | 2.38 | 1.00 (ref) |
|  | **ODD/CD** |  |  |  |
|  | Apgar score 1~3 | <6 | 0.51 | NA |
|  | Apgar score 4~6 | 32 | 0.60 | 1.14(0.80-1.61) |
|  | Apgar score 7~9 | 365 | 0.51 | 1.05(0.95-1.17) |
|  | Apgar score 10 | 4469 | 0.45 | 1.00 (ref) |

HR=Hazard Ratio, CI=Confidential Interval, ADHD=Attention Deficit Hyperactivity Disorder, ODD/CD=oppositional defiant disorder/conduct disorder

Cox models were adjusted for parental psychiatric history, maternal characteristics (parity, age at birth, smoking during pregnancy, highest education level, cohabitation with a partner, residence, birth country) and birth characteristics (participant’s sex, calendar year of birth, gestational age at birth and birth weight percentiles).
