# Supplementary material for: Five-Minute Apgar Score and the Risk of Mental Disorders During the First Four Decades of Life: A Nationwide Registry-Based Cohort Study in Denmark
Source: Front Med (Lausanne). 2022 Jan 14;8:796544. doi: 10.3389/fmed.2021.796544 (PMC8795588; doi:10.3389/fmed.2021.796544)
Supplement: Supplementary file 9 [file Table_9.DOCX]

**Table S9.** Hazard ratios of overall/specific mental disorders among individuals without neonatal brain lesions born after 1994 born with compromised 5-minute Apgar scores VS a score of 10.

| **exposures and outcomes** | | **No of events** | **rate per 1000 person years** | **HR (95% CI), adjusted** |
| --- | --- | --- | --- | --- |
| **Any mental disorder** | |  |  |  |
| Apgar score 1~3 | | 143 | 12.67 | 1.49 (1.26-1.76) |
| Apgar score 4~6 | | 683 | 11.34 | 1.30 (1.20-1.40) |
| Apgar score 7~9 | | 7432 | 9.01 | 1.11 (1.08-1.14) |
| Apgar score 10 | | 89110 | 7.63 | 1.00 (ref) |
| **Organic disorders** | |  |  |  |
| Apgar score 1~3 | | <6 | 0.18 | 5.18 (1.28-20.97) |
| Apgar score 4~6 | | <6 | 0.03 | 0.93 (0.23-3.75) |
| Apgar score 7~9 | | 37 | 0.04 | 1.38 (0.98-1.95) |
| Apgar score 10 | | 366 | 0.03 | 1.00 (ref) |
| **Substance use disorders** | |  |  |  |
| Apgar score 1~3 | | 8 | 2.26 | 1.48 (0.74-2.96) |
| Apgar score 4~6 | | 29 | 1.40 | 0.91 (0.63-1.31) |
| Apgar score 7~9 | | 367 | 1.35 | 0.91 (0.82-1.02) |
| Apgar score 10 | | 5915 | 1.55 | 1.00 (ref) |
| **Schizophrenia** | |  |  |  |
| Apgar score 1~3 | | <6 | 0.56 | 0.83 (0.21-3.34) |
| Apgar score 4~6 | | 13 | 0.63 | 0.98 (0.57-1.70) |
| Apgar score 7~9 | | 160 | 0.59 | 1.01 (0.86-1.18) |
| Apgar score 10 | | 2250 | 0.59 | 1.00 (ref) |
| **Mood disorders** | |  |  |  |
| Apgar score 1~3 | | <6 | 1.13 | 0.72 (0.27-1.91) |
| Apgar score 4~6 | | 40 | 1.93 | 1.27 (0.93-1.74) |
| Apgar score 7~9 | | 428 | 1.57 | 1.06 (0.96-1.17) |
| Apgar score 10 | | 5925 | 1.55 | 1.00 (ref) |
| **Neurotic disorders** | |  |  |  |
| Apgar score 1~3 | | 36 | 4.84 | 1.52 (1.09-2.10) |
| Apgar score 4~6 | | 137 | 3.29 | 1.04 (0.87-1.23) |
| Apgar score 7~9 | | 1766 | 3.17 | 1.08 (1.03-1.13) |
| Apgar score 10 | | 22775 | 2.91 | 1.00 (ref) |
|  | **OCD** |  |  |  |
|  | Apgar score 1~3 | 8 | 1.06 | 2.62 (1.30-5.25) |
|  | Apgar score 4~6 | 12 | 0.29 | 0.70 (0.40-1.24) |
|  | Apgar score 7~9 | 212 | 0.38 | 0.95 (0.83-1.10) |
|  | Apgar score 10 | 3017 | 0.38 | 1.00 (ref) |
| **Eating disorders** | |  |  |  |
| Apgar score 1~3 | | <6 | 0.33 | 0.90 (0.34-2.41) |
| Apgar score 4~6 | | 38 | 0.60 | 1.64 (1.19-2.26) |
| Apgar score 7~9 | | 325 | 0.38 | 1.12 (1.00-1.25) |
| Apgar score 10 | | 4178 | 0.35 | 1.00 (ref) |

HR=Hazard Ratio, CI=Confidential Interval, OCD= Obsessive-Compulsive Disorder

Cox models were adjusted for parental psychiatric history, maternal characteristics (parity, age at birth, smoking during pregnancy, highest education level, cohabitation with a partner, residence, birth country) and birth characteristics (participant’s sex, calendar year of birth, gestational age at birth and birth weight percentiles).

**Table S9. (Continued)** Hazard ratios of overall/specific mental disorders among individuals without neonatal brain lesions born after 1994 born with compromised 5-minute Apgar scores VS a score of 10.

| **exposures and outcomes** | | **No of events** | **rate per 1000 person years** | **HR (95% CI), adjusted** |
| --- | --- | --- | --- | --- |
| **Personality disorders** | |  |  |  |
| Apgar score 1~3 | | <6 | 1.13 | 1.83 (0.69-4.90) |
| Apgar score 4~6 | | 13 | 0.63 | 1.13 (0.65-1.95) |
| Apgar score 7~9 | | 146 | 0.54 | 1.04 (0.88-1.23) |
| Apgar score 10 | | 2179 | 0.57 | 1.00 (ref) |
| **Mental retardation** | |  |  |  |
| Apgar score 1~3 | | 31 | 2.61 | 4.62 (3.23-6.59) |
| Apgar score 4~6 | | 116 | 1.84 | 3.35 (2.78-4.04) |
| Apgar score 7~9 | | 644 | 0.75 | 1.62 (1.49-1.76) |
| Apgar score 10 | | 5018 | 0.42 | 1.00 (ref) |
| **Developmental disorders** | |  |  |  |
| Apgar score 1~3 | | 30 | 2.51 | 1.49 (1.04-2.13) |
| Apgar score 4~6 | | 149 | 2.36 | 1.39 (1.18-1.63) |
| Apgar score 7~9 | | 1556 | 1.82 | 1.13 (1.07-1.19) |
| Apgar score 10 | | 17140 | 1.42 | 1.00 (ref) |
|  | **Childhood autism** |  |  |  |
|  | Apgar score 1~3 | 14 | 1.16 | 1.63 (0.96-2.76) |
|  | Apgar score 4~6 | 61 | 0.96 | 1.41 (1.10-1.82) |
|  | Apgar score 7~9 | 605 | 0.70 | 1.12 (1.03-1.22) |
|  | Apgar score 10 | 6559 | 0.54 | 1.00 (ref) |
| **Behavioral disorders** | |  |  |  |
| Apgar score 1~3 | | 57 | 4.85 | 1.22 (0.94-1.58) |
| Apgar score 4~6 | | 300 | 4.82 | 1.18 (1.05-1.32) |
| Apgar score 7~9 | | 3508 | 4.16 | 1.09 (1.06-1.13) |
| Apgar score 10 | | 41459 | 3.49 | 1.00 (ref) |
|  | **ADHD** |  |  |  |
|  | Apgar score 1~3 | 32 | 3.36 | 1.22 (0.86-1.73) |
|  | Apgar score 4~6 | 180 | 3.49 | 1.24 (1.07-1.43) |
|  | Apgar score 7~9 | 1971 | 2.83 | 1.08 (1.03-1.13) |
|  | Apgar score 10 | 23351 | 2.38 | 1.00 (ref) |
|  | **ODD/CD** |  |  |  |
|  | Apgar score 1~3 | <6 | 0.52 | 1.07 (0.44-2.56) |
|  | Apgar score 4~6 | 30 | 0.57 | 1.09 (0.76-1.56) |
|  | Apgar score 7~9 | 362 | 0.51 | 1.05 (0.95-1.17) |
|  | Apgar score 10 | 4463 | 0.45 | 1.00 (ref) |

HR=Hazard Ratio, CI=Confidential Interval, ADHD=Attention Deficit Hyperactivity Disorder, ODD/CD=oppositional defiant disorder/conduct disorder

Cox models were adjusted for parental psychiatric history, maternal characteristics (parity, age at birth, smoking during pregnancy, highest education level, cohabitation with a partner, residence, birth country) and birth characteristics (participant’s sex, calendar year of birth, gestational age at birth and birth weight percentiles).
