# Supplementary material for: Five-Minute Apgar Score and the Risk of Mental Disorders During the First Four Decades of Life: A Nationwide Registry-Based Cohort Study in Denmark
Source: Front Med (Lausanne). 2022 Jan 14;8:796544. doi: 10.3389/fmed.2021.796544 (PMC8795588; doi:10.3389/fmed.2021.796544)
Supplement: Supplementary file 10 [file Data_Sheet_1.docx]

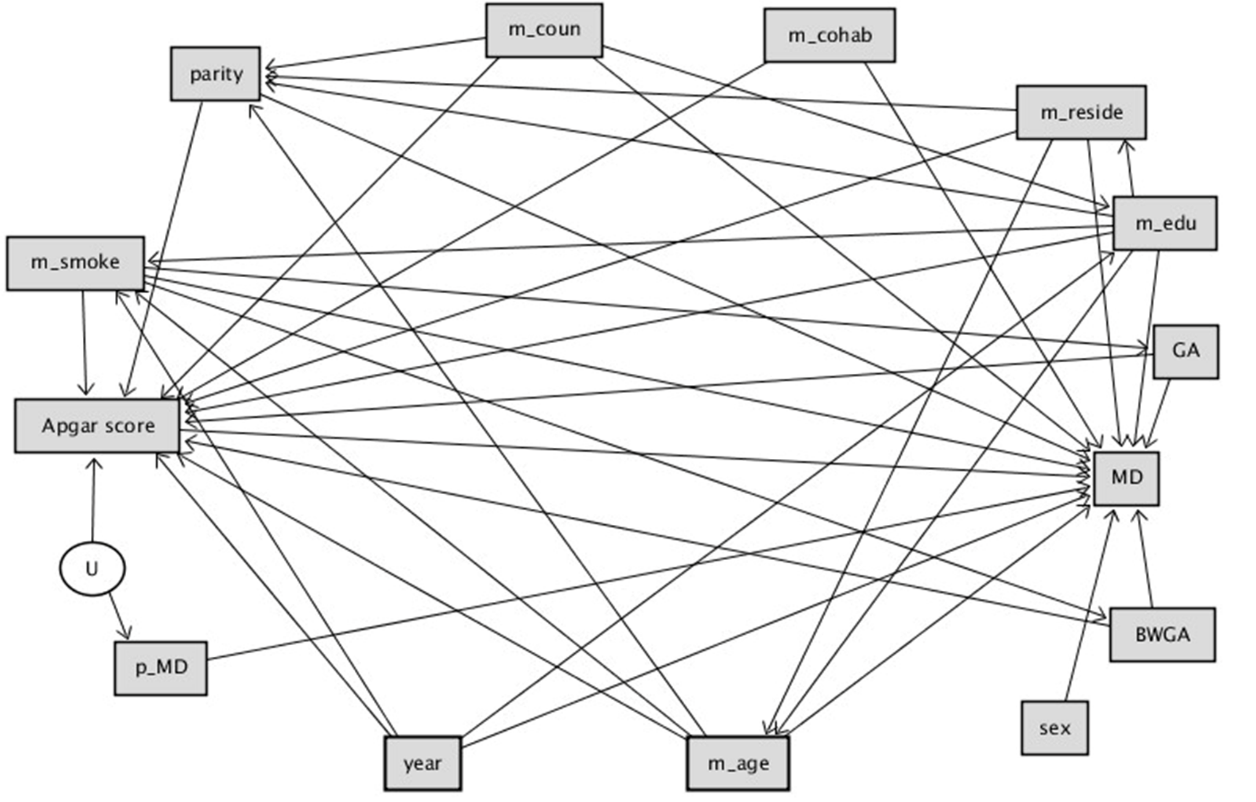


**Figure S1.** Directed Acyclic Graphs (DAGs) showing selection of covariates for confounding control

m_smoke: maternal smoking, m_coun: maternal country of birth, m_cohab: maternal cohabitation at childbirth, m_reside: maternal residence at childbirth,

m_deu: maternal education at childbirth, GA: gestational age at childbirth, MD: mental disorder of offspring, BWGA: birth weight for gestational age, m_age: maternal age at birth, year: calender year of birth, p_MD: paternal history of overall mental disorders, U: unmeasured variable





**Figure S2.** Hazard ratios of overall mental disorder in relation to Apgar score at 5 minutes across gestational age strata among individuals in childhood. Cox models were adjusted for parental psychiatric history, maternal characteristics (parity, age at birth, smoking during pregnancy, highest education level, cohabitation with a partner, residence, birth country,) and birth characteristics (participant’s sex, calendar year of birth and birth weight percentiles).





**Figure S3.** Hazard ratios of overall mental disorder in relation to Apgar score at 5 minutes across birth weight percentile strata among individuals in childhood.

Cox models were adjusted for parental psychiatric history, maternal characteristics (parity, age at birth, smoking during pregnancy, highest education level, cohabitation with a partner, residence, birth country,) and birth characteristics (participant’s sex, calendar year of birth and gestational age).
